# Systematic analysis of global health research funding in Canada, 2000–2016

**DOI:** 10.17269/s41997-019-00247-8

**Published:** 2019-11-06

**Authors:** Steven J. Hoffman, Elliot Gunn, Susan Rogers Van Katwyk, Stephanie Nixon

**Affiliations:** 1 Global Strategy Lab, Dahdaleh Institute for Global Health Research, Faculty of Health and Osgoode Hall Law School, York University, Toronto, Ontario Canada; 2 Department of Health Research Methods, Evidence & Impact and McMaster Health Forum, McMaster University, Hamilton, Ontario Canada; 3 Department of Global Health & Population, Harvard T.H. Chan School of Public Health, Harvard University, Boston, MA USA; 4 School of Epidemiology & Public Health, University of Ottawa, Ottawa, Ontario Canada; 5 Department of Physical Therapy, University of Toronto, Toronto, Ontario Canada; 6 Dalla Lana School of Public Health, University of Toronto, Toronto, Ontario Canada; 7 International Centre for Disability and Rehabilitation, University of Toronto, Toronto, Ontario Canada

**Keywords:** Global health, Research, Capacity building, Funding, Research support as topic, Canada, Santé mondiale, Recherche, Renforcement des capacités, Financement, Soutien financier à la recherche comme sujet, Canada

## Abstract

**Objectives:**

Considering recent shifts in global funding landscapes, this study analyzes Canada’s long-term global health research funding trends in the hope of informing a new Canadian global health research strategy. Examining past investments can help prioritize limited future resources to either build on Canada’s existing strengths or fill gaps where needed, while simultaneously informing the investments of research funders in other countries.

**Methods:**

Administrative data were analyzed covering all 1584 global health research grants awarded by the Canadian Institutes of Health Research (CIHR) to 927 unique principal investigators from 2000 to 2016, totalling C$341 million. Existing metadata associated with each grant was supplemented by additional qualitative coding. Descriptive time-series analyses of global health research grant data were conducted using various measures related to each grant’s recipient (e.g., province, university, sex, distribution) and subject matter (e.g., research theme, area, focus).

**Results:**

CIHR’s total annual global health research funding increased sharply from $3.6 million in FY2000/2001 to $30.3 million in FY2015/2016, with the largest share of research funding now focused on health equity—representing nearly 50% of CIHR’s global health research funding. Past grants have concentrated on infectious disease and public health research. One third of CIHR’s global health grant funding went to 20 principal investigators. Only 42.2% of global health research funding came from CIHR’s open investigator-driven competitions, with the rest coming from strategic priority-driven competitions.

**Conclusion:**

Global health research has seen steady increases in funding from CIHR’s open competitions when preceded by investment in strategic competitions, which suggests the level of a national research funding agency’s strategic investments in global health research may determine the size of the field in their country. The greatest concentration of past investment lies in health equity research, followed by infectious disease research. Future analyses of research funding would benefit from an internationally accepted keyword classification scheme and more granular administrative data.

**Electronic supplementary material:**

The online version of this article (10.17269/s41997-019-00247-8) contains supplementary material, which is available to authorized users.

## Résumé

**Objectifs:**

Étant donné les changements récents dans la conjoncture mondiale du financement, nous analysons les tendances à long terme du financement de la recherche en santé mondiale du Canada dans l’espoir d’éclairer une nouvelle stratégie canadienne de recherche en santé mondiale. L’examen des investissements passés peut contribuer à déterminer s’il faut consacrer les ressources futures limitées à miser sur les forces existantes du Canada ou à combler les lacunes si nécessaire, tout en jetant un éclairage sur les investissements dans la recherche des bailleurs de fonds d’autres pays.

**Méthode:**

Nous avons analysé les données administratives des 1 584 subventions de recherche en santé mondiale, d’une valeur totale de 341 M$ CA, octroyées par les Instituts de recherche en santé du Canada (IRSC) à 927 chercheurs en chef individuels entre 2000 et 2016. Les métadonnées existantes associées à chaque subvention ont été complétées par un codage qualitatif. Des analyses descriptives des séries chronologiques des données sur les subventions de recherche en santé mondiale ont été effectuées à l’aide de divers indicateurs, liés à chaque bénéficiaire de subventions (p. ex. province, université, sexe, répartition) et au sujet traité (p. ex. sujet, domaine, axe de recherche).

**Résultats:**

Le financement annuel total de la recherche en santé mondiale par les IRSC a connu une forte augmentation, passant de 3,6 M$ en 2000-2001 à 30,3 M$ en 2015-2016, la plus grande proportion du financement de la recherche étant maintenant consacrée à l’équité en santé – qui représente près de 50 % du financement de la recherche en santé mondiale des IRSC. Les subventions passées se concentraient sur la recherche sur les maladies infectieuses et la recherche en santé publique. Le tiers du financement des subventions de recherche en santé mondiale des IRSC est allé à 20 chercheurs en chef. Seulement 42,2 % du financement de la recherche en santé mondiale est venu des concours de subventions ouverts des IRSC, et le reste, des concours stratégiques priorisés.

**Conclusion:**

Le financement de la recherche en santé mondiale venant des concours ouverts des IRSC augmente de façon soutenue quand il est précédé par des investissements dans les concours stratégiques, ce qui est signe que le niveau d’investissements stratégiques dans la recherche en santé mondiale d’un organisme national de financement de la recherche peut déterminer l’ampleur du champ dans son pays. La plus forte concentration d’investissements passés est allée à la recherche sur l’équité en santé, suivie par la recherche sur les maladies infectieuses. Il serait bon que les futures analyses du financement de la recherche utilisent un système de classification des mots-clés mondialement accepté et des données administratives plus granulaires.

## Introduction

Budget constraints and shifting political priorities have long posed challenges for global health research funding. Global health research is defined as not only supporting health research in low- and middle-income countries (LMICs), but also focusing on “health, health-system, health inequities, and health policy challenges facing populations living in conditions of vulnerability” in both LMICs and high-income countries (HICs) (Global health research 2017). In the wake of Brexit and the 2016 United States (US) election, a new uncertainty has set in about whether the United Kingdom (UK) and the US will maintain their historic levels of support for global health research initiatives (Greenwood 2018). Global development assistance for health has grown by only 1.2% annually since 2010, a sharp drop from the 11.3% annual increases seen throughout the 2000s, and continued limitations on growth are expected (Dieleman et al. 2016). Globally, public funding of global health research has declined since the global financial crisis of 2008 (Chapman et al. 2016); existing analyses show that only a small percentage of global health research funds are allocated towards epidemiology and health policy and systems research, which is context-specific research that is especially important for LMICs (Abimbola et al. 2017). Many observers worry about continued reductions in research funding opportunities for global health, as populist pressures may encourage countries to shift resources towards domestic priorities (Morrison 2012). This global shortfall runs counter to the “convergence plan” of *The Lancet*’s Global Health 2035 Commission, which calls for doubling research funding for diseases that disproportionately affect LMICs from US$3 billion to US$6 billion per year so that health status in LMICs can catch up to HICs within a generation (Ramesh 2013). In light of these challenges, other countries, even if smaller in influence and capacity, will increasingly be shaping global health research priorities alongside existing major funders.

In this uncertain global funding climate, it is helpful to probe the role that “middle powers” like Canada can play in global health research (Nixon et al. 2018). The optimistic view is that Canada has long led efforts to shape the global health agenda and has supported significant developments in global health research. For example, researchers in Canada conducted the world’s largest randomized trial on breastfeeding interventions, which influenced World Health Organization (WHO) guidelines (Patel et al. 2013). Evidence-based medicine was introduced into clinical practice in the 1990s by Canadian doctors at McMaster University (Smith and Rennie 2014). The University of Waterloo’s International Tobacco Control Policy Evaluation Project was the first international research program to evaluate the WHO Framework Convention on Tobacco Control’s provisions (International Tobacco Control Policy Evaluation Project n.d.). The Canadian-developed Ebola vaccine was the first to receive regulatory approval (Beaudet 2015).

Yet, Canada has not been immune to funding challenges. As a middle power, Canada faces limitations in its ability to invest in all areas of global health research. The combination of the 2008 global financial crisis and the emergence of global health threats led to reforms and shifts in Canada’s global health research funding environment, with increased pressure on both funders and grantees to prove domestic research impacts for Canadians (Plamondon et al. 2017). Given resource limitations, such impact is probably more likely to be achieved—or at least to be more easily measured—if future strategic research funding opportunities are focused on a smaller range of global health issues or allocated to fill essential gaps.

Over the last decade, global health research funding for Canadian researchers has become increasingly concentrated within the Canadian Institutes of Health Research (CIHR), the Canadian federal government’s funding agency for health research. CIHR is one of three primary global health research funders in Canada, along with the International Development Research Centre (IDRC) and Grand Challenges Canada (GCC). Collectively, IDRC and GCC fund more global health research than CIHR; at their peaks in 2012/2013, IDRC invested C$16 million and GCC invested C$54 million in global health research (Plamondon et al. 2017). But the vast majority of these investments have appropriately supported LMIC researchers—not Canadian researchers—given these funds come from Canada’s official development assistance (ODA) budget. CIHR funding became even more important for Canadian global health researchers when Canada’s Social Sciences and Humanities Research Council (SSHRC) stopped funding health research in 2009. (SSHRC started to accept proposals for health social science research in 2016 on a limited basis (Subject matter eligibility 2017).

CIHR has listed global health research among its priorities since its creation in 2000. Since then, CIHR sponsored the Global Health Research Initiative (GHRI), which was a partnership between CIHR, IDRC, the former Canadian International Development Agency, and Health Canada (Global health 2013). CIHR collaborated with international health research funders to form the Global Alliance for Chronic Diseases (GACD), helped launch GCC, stewarded the Government of Canada’s contribution to the Canada Gairdner Award for Global Health, created an Emerging Health Threats program that funded research during recent Ebola and Zika outbreaks, and co-funded the seven-year Innovating Maternal and Child Health in Africa (IMCHA) funding program with IDRC (Ramesh 2013).

As Canada is currently refreshing its global health research strategy, developing its action plan for the Sustainable Development Goals, and implementing a new feminist international development policy, an analysis of long-term global health research funding trends can inform and provide historical context for these efforts. This study presents such an analysis focused on CIHR, which is unique among Canada’s three primary global health research funders because it supports all kinds of health research and is by far the largest source of funding for such research conducted by researchers based at Canadian academic institutions. (GCC’s annual investment in global health research is larger when combining its domestic and foreign funding activities (Plamondon et al. 2017).)

Specifically, we aimed to gain a more comprehensive understanding of what kinds of global health research have been funded in Canada by examining a novel administrative dataset of global health research funding made available by CIHR. We identified, analyzed, and segmented long-term funding trends by various measures related to each grant’s recipient (e.g., province, university, sex, distribution) and subject matter (e.g., research theme, area, focus). To date, there has been only limited formal analysis of global health research funding in Canada, even though it provides valuable insight into global health research agenda setting. Spanning 15 years and representing C$341 million invested, these data let us identify shifts, specializations, and gaps in research focus; distributional patterns across researchers; and potential areas where CIHR and other global health research funders internationally may wish to invest either to build on existing strengths or fill essential gaps.

## Methods

### Identification of global health research grants

We analyzed a novel administrative dataset covering 15 fiscal years (FYs) of CIHR’s global health research funding (see Web Appendix 1 for the full dataset). Potential global health research grants were identified annually through systematic keyword searches of CIHR’s research funding database (see Web Appendix 2 for the most recent search string). Each grant in the administrative dataset was individually validated by staff at CIHR’s Institute of Population and Public Health (FY2000/2001–2014/2015) and CIHR’s Strategic Partnerships and International Relations Team (FY2015/2016–present) as being relevant to global health. An additional validation review of all grants was conducted for this study by one researcher (EG) to minimize false positives.

### Dataset development and analysis

The final dataset includes basic administrative metadata collected routinely upon submission of grant applications, such as unique applicant numbers, co-applicants, program names, host institutions, and province. The dataset also includes information related to the grant application such as keywords, abstracts, research themes, research areas, program funding types, and amounts disbursed by fiscal year. We calculated adjustments for inflation using Statistics Canada’s Consumer Price Index to allow us to set all monetary figures to 2015 Canadian dollar equivalents as appropriate. One researcher (EG) filled in missing information for two categories in the dataset—research theme and area—by manually coding and classifying the grants using the information provided in the grant application’s abstract and internet searches when required.

The coding definitions for each category can be found in Table 1. In summary, *research theme* corresponds with CIHR’s four health research themes: (1) biomedical research, (2) clinical research, (3) health services research, and (4) population health research. *Institute research area* matches the topical mandates of CIHR’s 13 funding institutes (whether or not grants were actually funded by those institutes)—with each grant allocated to a single, primary institute research area. *Funding by province* consists of 10 provinces, including an unspecified category for when the province was not specified. *Funding by program type* refers to grants versus awards, an important technical distinction; *grants* generally cover a broader range of research costs including direct research costs, training of researchers, and knowledge translation activities, whereas *awards* include direct salary support to individual research personnel and stipends for individual research trainees. CIHR funding is also designated as either “open” or “strategic”. *Open funding* refers to investigator-driven projects proposed by researchers without any direction on funding priorities. *Strategic funding* refers to priority-driven initiatives created by CIHR on particular topics that are deemed to be of strategic importance to Canada.

**Table 1:** Definitions used in qualitative coding

Category	Definition
**Research theme, corresponding with CIHR’s four health research themes or pillars**	
Biomedical research	Research with the goal of understanding normal and abnormal human functioning, at the molecular, cellular, organ system, and whole body levels, including development of tools and techniques to be applied for this purpose; developing new therapies or devices that improve health or the quality of life of individuals, up to the point where they are tested on human subjects. Biomedical research may also include studies on human subjects that do not have a diagnostic or therapeutic orientation.
Clinical research	Research with the goal of improving the diagnosis and treatment (including rehabilitation and palliation) of disease and injury; improving the health and quality of life of individuals as they pass through normal life stages. Clinical research usually encompasses research on, or for the treatment of, patients.
Health services research	Research with the goal of improving the efficiency and effectiveness of health professionals and the health care system, through changes to practice and policy. Health services research is a multidisciplinary field of scientific investigation that studies how social factors, financing systems, organizational structures and processes, health technologies, and personal behaviours affect access to health care, the quality and cost of health care, and, ultimately, health and well-being.
Population health research	Research with the goal of improving the health of populations, or of defined subpopulations, through a better understanding of the ways in which social, cultural, environmental, occupational, and economic factors determine health status.
**Institute research area, corresponding with CIHR’s 13 Institutes**	
Aging	Research that promotes healthy aging and addresses causes, prevention, screening, diagnosis, treatment, support systems, and palliation for a wide range of conditions associated with aging.
Cancer research	Research on the prevention and treatment of cancer and on improving the health and quality of life of cancer patients.
Circulatory and respiratory health	Research into the causes, mechanisms, prevention, screening, diagnosis, treatment, support systems, and palliation for a wide range of conditions associated with the heart, lung, brain (stroke), blood, blood vessels, critical care, and sleep.
Gender and health	Research on the influence of gender and sex on the health of women and men throughout life and the application of these research findings to identify and address pressing health challenges.
Genetics	Research on the human and model genomes and on all aspects of genetics, basic biochemistry, and cell biology related to health and disease, including the translation of knowledge into health policy and practice, and the societal implications of genetic discoveries.
Health services and policy research	Research designed to improve the way health care services are organized, regulated, managed, financed, paid for, used, and delivered, in the interest of improving the health and quality of life of all Canadians.
Human development, child, and youth health	Research that ensures the best start in life for all Canadians and the achievement of their potential for optimal growth and development.
Indigenous Peoples’ health	Research to improve and promote the health of Indigenous Peoples in Canada.
Infection and immunity	Research in the areas of infectious disease and the body’s immune system.
Musculoskeletal health and arthritis	Research to enhance active living, mobility and movement, and oral health, and that addresses causes, prevention, screening, diagnosis, treatment, support systems, and palliation for a wide range of conditions related to bones, joints, muscles, connective tissue, skin, and teeth.
Neurosciences, mental health, and addiction	Research to enhance mental health, neurological health, vision, hearing, and cognitive functioning and to reduce the burden of related disorders through prevention strategies, screening, diagnosis, treatment, support systems, and palliation.
Nutrition, metabolism, and diabetes	Research to enhance health in relation to diet, digestion, excretion, and metabolism, and to address causes, prevention, screening, diagnosis, treatment, support systems, and palliation for a wide range of conditions and problems associated with hormone, digestive system, kidney, and liver function.
Population and public health	Research into the complex biological, social, cultural, and environmental interactions that determine the health of individuals, communities, and global populations.
**Research focus**	
Globalization	Explaining patterns of health, health equity, and well-being within a global context or as shaped by global economic, social, cultural, environmental, or political factors.
Health equity	Improving the health of populations facing conditions of marginalization, such as people living with poverty in LMICs and Indigenous Peoples in multiple international settings.
Neglected conditions	Tackling the circumstances, conditions, and diseases that disproportionately affect disadvantaged populations who are excluded from markets and society.
Transnational risks	Addressing health threats, opportunities, determinants or solutions that transcend political boundaries.
Not specified	The titles and abstracts of grant applications did not provide enough context for coding the research focus.
**Program type**	
Award program	Direct salary support to individual research personnel or stipend support to individual research trainees.
Grant program	Support for the direct costs of research projects, including for the training of researchers and/or activities that support the translation of research findings, conducted by either an investigator working alone or by a group of investigators working together.
Open competition	Investigator-driven research competitions with projects, initiated by individuals or teams and without restrictions on topic.
Strategic competition	Priority-driven research competitions to build capacity and/or support research excellence in specific areas of health research that are deemed to be of strategic importance to the country.

We additionally analyzed how global infectious disease research funding has evolved over time. We looked at specific global health threats that have been widely discussed in the news media: HIV/AIDS, Ebola, severe acute respiratory syndrome (SARS), H1N1 influenza, and tuberculosis. Distribution of funds was analyzed by calculating the share of total research funding held by the 20 principal investigators (PIs) who received the most global health research funding overall. Data on PIs’ sex were not available through the original administrative dataset but were retrieved from CIHR’s Funding Analytics Team through a special access request.

We defined four *foci* of global health research that we believe are fully encompassing of the field and qualitatively coded grants into each focus based on the grant application’s title and abstract. The four foci were:

1. *Globalization*, which refers to explaining patterns of health, health equity, and well-being within a global context or as shaped by global economic, social, cultural, environmental, or political factors;
2. *Health equity*, which is concerned with improving the health of populations facing conditions of marginalization, such as people living with poverty in LMICs and Indigenous Peoples in multiple international settings;
3. *Neglected conditions*, which tackles the circumstances, conditions, and diseases that disproportionately affect disadvantaged populations who are excluded from markets and society, including biomedical research on these diseases and conditions;
4. *Transnational risks*, which address health threats, opportunities, determinants, or solutions that transcend political boundaries.

When grants pertained to multiple foci, they were coded per the single primary focus that fit best. Grants were coded as “not specified” when the abstracts of grant applications either were not available or did not provide enough information to code the focus.

We conducted descriptive time-series analyses of available data. We looked at trends over time in eight categories: research theme, institute research area, research focus, geographic distribution, program type, PI distribution, sex, and infectious diseases.

## Results

The dataset captures C$340,833,742 in funding across 1584 different grants and 927 PIs (Table 2). Data validation checks identified only one false positive in the original administrative dataset, which was removed. Total annual funding has grown rapidly by 742%, from C$3.6 million in FY2000/2001 to C$30.3 million in FY2015/2016 (in 2015 Canadian dollars).

**Table 2:** CIHR funding for global health research by various measures related to each grant’s recipient and subject matter

Categories/fiscal year	2000/2001	2001/2002	2002/2003	2003/2004	2004/2005	2005/2006	2006/2007	2007/2008	2008/2009	2009/2010	2010/2011	2011/2012	2012/2013	2013/2014	2014/2015	2015/2016	Total
**Research theme**																	$340,829,872
Biomedical (40.1%)	$2,497,370	$4,034,136	$4,297,848	$5,044,831	$5,254,433	$7,285,730	$8,520,410	$10,163,394	$11,463,202	$12,859,270	$14,043,913	$11,071,351	$11,307,136	$10,684,967	$10,235,703	$8,053,635	$136,817,328
Clinical (14.2%)	$556,039	$1,418,950	$1,668,013	$1,136,653	$1,448,252	$1,226,349	$2,622,185	$3,660,307	$4,971,014	$4,367,131	$5,791,882	$3,954,212	$4,545,706	$4,240,470	$3,821,045	$2,797,971	$48,226,180
Health services (11.3%)	$409,384	$396,596	$1,190,242	$2,114,667	$1,613,007	$1,391,096	$1,483,290	$2,059,707	$3,506,540	$3,203,045	$2,620,046	$2,811,811	$3,584,984	$4,197,447	$3,970,401	$3,854,289	$38,406,554
Population health (34.2%)	$0	$355,310	$1,960,619	$6,114,894	$5,576,162	$4,232,731	$6,498,903	$8,068,315	$7,451,003	$7,931,919	$6,381,360	$9,523,155	$12,100,666	$12,238,866	$12,503,847	$15,558,214	$116,495,964
Not specified (0.3%)	$131,235	$95,170	$66,769	$34,970	$5,635	$42,960	$161,180	$151,950	$148,141	$25,926	$0	$0	$0	$10,309	$9,600	$0	$883,845
**Research category**																	$340,833,742
Globalization (10.8%)	$0	$37,864	$429,554	$230,457	$169,050	$229,024	$1,956,992	$3,207,084	$4,518,022	$5,110,755	$4,435,360	$3,480,038	$2,760,818	$3,372,019	$3,171,459	$3,836,475	$36,944,972
Health equity (47.4%)	$127,229	$1,392,060	$2,892,816	$7,557,468	$6,763,704	$5,970,696	$8,238,658	$10,306,316	$11,673,681	$10,519,006	$11,106,701	$13,473,596	$18,001,926	$18,121,802	$18,133,981	$17,255,091	$161,534,730
Neglected conditions (27.5%)	$2,344,199	$3,850,528	$4,109,595	$4,227,811	$4,495,269	$5,381,061	$6,110,456	$7,219,115	$7,936,511	$8,856,427	$8,647,425	$6,244,419	$6,738,218	$6,193,344	$5,710,086	$5,540,937	$93,605,400
Transnational risks (6.3%)	$365,029	$647,329	$886,200	$1,344,334	$1,425,443	$1,635,745	$1,721,999	$1,515,187	$1,612,397	$1,637,072	$1,728,415	$1,112,824	$1,480,828	$1,528,752	$1,410,063	$1,538,507	$21,590,124
Not specified (8.0%)	$757,572	$372,381	$865,326	$1,085,945	$1,044,023	$962,341	$1,257,862	$1,855,970	$1,799,290	$2,264,032	$2,919,300	$3,049,654	$2,550,461	$2,156,142	$2,125,120	$2,093,099	$27,158,517
**Institute research area**																	$339,946,636
Aging (1.7%)	$0	$0	$0	$20,283	$0	$0	$0	$112,476	$202,076	$354,661	$439,161	$682,998	$961,281	$1,103,704	$1,063,587	$891,681	$5,831,908
Cancer research (1.0%)	$28,571	$25,281	$4,220	$0	$0	$0	$18,857	$56,822	$184,129	$266,117	$463,616	$725,851	$652,641	$534,351	$395,052	$178,542	$3,534,049
Circulatory and respiratory health (5.0%)	$52,750	$0	$241,127	$0	$0	$32,044	$106,654	$692,843	$1,637,655	$2,104,235	$2,137,519	$1,579,553	$1,697,386	$1,869,820	$1,907,781	$2,842,062	$16,901,428
Gender and health (2.7%)	$0	$0	$221,164	$252,461	$46,543	$247,247	$239,976	$592,115	$745,057	$889,881	$628,225	$1,169,995	$1,227,660	$1,014,809	$1,069,495	$985,410	$9,330,039
Genetics (4.2%)	$184,577	$297,107	$336,170	$291,717	$332,137	$412,999	$615,415	$703,968	$1,367,109	$2,144,930	$2,300,718	$2,051,835	$1,514,764	$1,017,999	$324,659	$283,572	$14,179,678
Health services and policy research (5.7%)	$54,967	$46,061	$335,750	$848,105	$779,601	$945,207	$886,064	$853,547	$867,131	$866,087	$732,513	$1,308,692	$2,770,252	$2,919,628	$2,446,078	$2,678,507	$19,338,192
Human development, child, and youth health (4.9%)	$0	$68,541	$213,772	$194,603	$240,715	$263,410	$1,704,897	$2,533,183	$2,930,304	$1,944,526	$2,808,290	$1,234,537	$952,377	$669,081	$595,141	$412,891	$16,766,269
Indigenous Peoples’ health (0.7%)	$0	$0	$0	$583,332	$204,813	$115,872	$129,265	$160,459	$181,090	$294,708	$157,571	$59,129	$102,861	$40,550	$121,443	$185,000	$2,336,094
Infection and immunity (39.7%)	$2,997,396	$4,289,790	$4,829,596	$6,854,185	$8,075,907	$7,368,653	$8,925,144	$10,541,253	$10,685,505	$11,300,545	$10,164,255	$8,095,819	$10,242,422	$11,115,735	$10,625,887	$8,905,774	$135,017,866
Musculoskeletal health and arthritis (1.1%)	$0	$0	$0	$0	$0	$0	$0	$36,071	$197,006	$247,427	$356,833	$350,907	$480,004	$472,909	$780,932	$653,533	$3,575,621
Neurosciences, mental health, and addiction (3.9%)	$0	$0	$120,424	$683,886	$647,956	$688,198	$520,783	$911,884	$705,269	$576,369	$1,313,799	$1,488,799	$1,726,988	$1,542,584	$1,238,542	$967,488	$13,132,970
Nutrition, metabolism, and diabetes (3.8%)	$144,531	$322,676	$425,404	$549,214	$671,259	$832,121	$1,215,709	$1,103,585	$1,231,435	$1,129,167	$1,131,168	$857,103	$741,989	$758,854	$951,233	$939,723	$13,005,170
Population and public health (25.6%)	$0	$1,138,788	$2,376,616	$4,133,258	$2,877,260	$3,226,082	$4,782,106	$5,651,245	$6,475,100	$6,242,712	$6,203,534	$7,755,310	$8,467,865	$8,301,727	$9,025,823	$10,339,926	$86,997,352
**Province**																	$340,839,984
Alberta (3.8%)	$173,822	$215,998	$367,408	$820,035	$506,601	$625,141	$896,376	$1,045,214	$1,597,324	$1,261,565	$1,078,417	$912,583	$732,994	$857,754	$996,963	$833,926	$12,922,120
British Columbia (12.8%)	$308,877	$302,214	$410,082	$1,284,299	$908,381	$1,429,838	$1,036,039	$2,298,104	$2,848,018	$3,397,314	$2,849,658	$3,735,080	$5,410,317	$5,425,961	$5,182,314	$5,002,492	$41,828,990
Manitoba (7.6%)	$64,185	$1,226,029	$1,559,980	$1,552,388	$1,417,786	$2,154,576	$2,097,472	$1,972,797	$1,638,921	$1,858,867	$1,965,487	$1,706,378	$1,859,518	$1,792,215	$1,745,976	$1,304,157	$25,916,730
New Brunswick (0.0%)	$0	$0	$0	$0	$0	$0	$29,010	$0	$0	$0	$0	$0	$26,007	$0	$0	$10,000	$65,017
Newfoundland and Labrador (0.0%)	$0	$0	$0	$9,970	$0	$0	$0	$0	$0	$0	$0	$0	$0	$0	$0	$0	$9,970
Nova Scotia (1.7%)	$54,967	$64,364	$55,394	$175,367	$238,422	$58,236	$205,198	$216,008	$401,096	$479,856	$623,253	$694,997	$684,614	$635,951	$610,024	$535,278	$5,733,023
Ontario (30.7%)	$512,346	$625,886	$2,062,972	$2,923,301	$2,075,663	$2,512,111	$5,458,224	$7,030,274	$8,874,011	$9,417,357	$9,623,004	$8,593,534	$10,990,982	$11,413,364	$11,355,002	$11,168,674	$104,636,706
Prince Edward Island (0.1%)	$0	$0	$0	$0	$0	$0	$0	$0	$13,869	$55,332	$95,086	$0	$19,505	$0	$0	$0	$183,792
Quebec (30.8%)	$1,855,989	$2,691,882	$3,026,933	$5,152,675	$6,132,371	$4,501,782	$6,229,420	$8,047,970	$9,410,522	$9,009,548	$10,541,019	$9,286,091	$8,139,336	$7,553,219	$6,457,185	$6,937,499	$104,973,440
Saskatchewan (1.6%)	$197,546	$218,075	$343,556	$738,452	$430,136	$618,971	$559,324	$882,026	$464,421	$481,483	$153,204	$92,389	$62,083	$91,496	$130,611	$100,833	$5,564,608
Not specified (11.4%)	$426,295	$955,715	$1,357,166	$1,789,527	$2,188,129	$2,278,211	$2,774,906	$2,611,281	$2,291,719	$2,425,971	$1,908,072	$2,339,476	$3,613,137	$3,602,100	$4,072,633	$4,371,250	$39,005,587
**Program type: grant/award**																	$340,839,984
Award program (21.9%)	$837,150	$1,709,847	$2,334,391	$3,081,058	$3,379,515	$4,153,487	$4,626,011	$5,032,102	$5,409,271	$6,145,917	$5,531,094	$5,288,366	$6,520,194	$6,534,805	$6,989,867	$7,168,937	$74,742,012
Grant programs (78.1%)	$2,756,878	$4,590,315	$6,849,101	$11,364,957	$10,517,975	$10,025,379	$14,659,956	$19,071,571	$22,130,630	$22,241,375	$23,306,107	$22,072,163	$25,018,298	$24,837,254	$23,560,841	$23,095,172	$266,097,972
**Program type: open/strategic**																	$340,839,984
Open (42.2%)	$1,588,927	$2,566,078	$2,386,147	$3,858,793	$5,679,095	$4,569,985	$7,895,492	$11,152,482	$11,626,822	$11,963,252	$13,569,212	$12,083,370	$13,281,792	$13,289,269	$13,511,205	$14,913,620	$143,935,540
Strategic (57.8%)	$2,005,101	$3,734,084	$6,797,345	$10,587,222	$8,218,395	$9,608,882	$11,390,476	$12,951,190	$15,913,079	$16,424,039	$15,267,989	$15,277,159	$18,256,700	$18,082,790	$17,039,504	$15,350,489	$196,904,444
**Total by FY**	$3,594,028	$6,300,162	$9,183,491	$14,446,015	$13,897,490	$14,178,867	$19,285,968	$24,103,672	$27,539,901	$28,387,291	$28,837,201	$27,360,529	$31,538,492	$31,372,059	$30,550,709	$30,264,109	

### Research theme

**Fig. 1:**
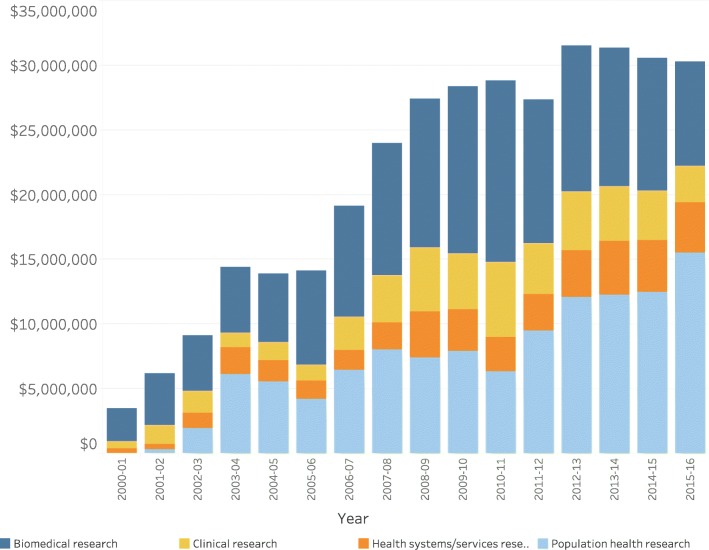
CIHR funding for global health research by research theme, 2000–2016

Approximately two thirds of overall funding has supported two research themes: biomedical research and population health research. Population health research has been the fastest growing research theme, starting from zero funding in FY2000/2001 to C$15.6 million in FY2015/2016 (Fig. 1). The share of funding for biomedical research has dipped in recent years—although it still represents more than one third of CIHR’s total global health research funding in FY2015/2016.

### Institute research area

**Fig. 2:**
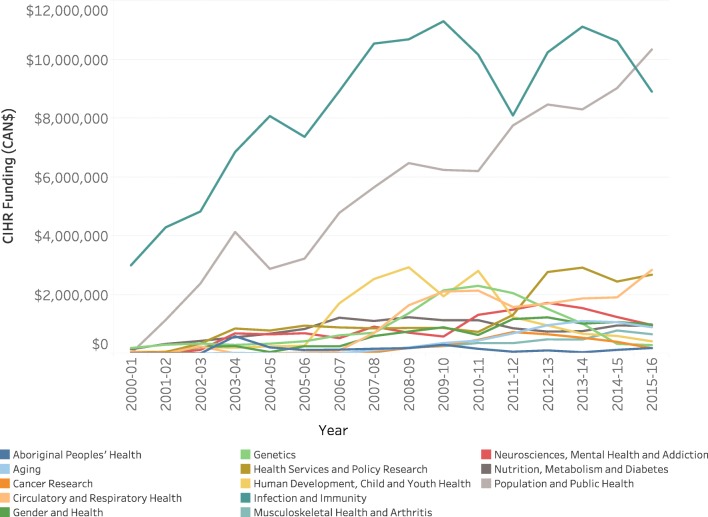
CIHR funding for global ealth research by Institute research area, 2000–2016

The top five institute research areas cumulatively from highest to lowest are infection and immunity (C$135 million, 39.6%); population and public health (C$87 million, 25.5%); health services and policy research (C$19 million, 5.7%); circulatory and respiratory health (C$17 million, 5.0%); and human development, child, and youth health (C$17 million, 4.9%) (Fig. 2). Funding is heavily concentrated in the first two areas; in fact, two thirds of Canadian global health research funding has supported research in either infectious diseases or public health. Conversely, only 0.7% and 1.0% of global health research funding have supported research for Indigenous Peoples’ health and for cancer, respectively. This result shows there are relatively few CIHR-funded studies with Indigenous People and on cancer that involve populations in LMICs or facing conditions of marginalization across multiple international settings.

### Research focus

**Fig. 3:**
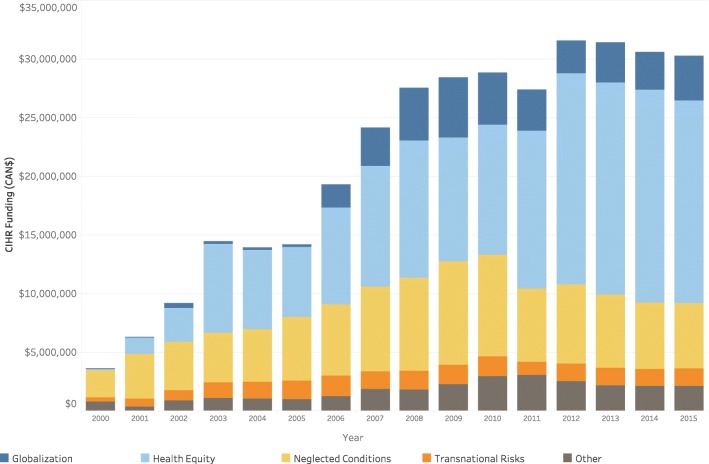
CIHR funding for global health research by research focus, 2000–2016

The largest research focus of CIHR’s global health research grants has been health equity, representing 47.4% or C$161.5 million (Fig. 3). This focus receives almost twice as much funding as the next largest research focus, neglected conditions (C$93.6 million, 27.5%). Health equity is also the fastest growing focus with annual funding in FY2015/2016 representing a 13,462% increase since FY2000/2001. Funding for transnational risks has remained steady over 15 fiscal years, while globalization funding increased sharply after FY2005/2006.

Global infection and immunity research has received the most annual funding until recently when funding for global population and public health research overtook it. Global health services and policy research increased sharply after FY2011/2012, and global circulatory and respiratory health research increased after FY2007/2008. Global human development, child, and youth health research decreased steadily since its peak in FY2010/2011, possibly because of an emphasis starting at that time on maternal, newborn, and child health services research which was classified as health services and policy research (instead of as research on human development, child, and youth health).

### Geographic distribution

Thirty-one percent of CIHR’s global health research funding was awarded to PIs based in each of Ontario and Quebec, and 14 of the 20 highest funded PIs are based in these provinces. Only $9970 (< 0.01%), $65,017 (0.02%), and $183,792 (0.1%) of CIHR’s global health research funding has been awarded to PIs based in Newfoundland and Labrador, New Brunswick, and Prince Edward Island, respectively.

### Program type

Across all areas of research, CIHR’s funds are mostly disbursed through grant programs (78.1%) as opposed to award programs (21.9%). CIHR’s open investigator-driven programs (i.e., Project and Foundation Scheme grants) account for approximately 70% of CIHR’s annual research funding (Design discussion document 2012). Within global health research, open programs are responsible for only 42.2% of CIHR’s funding, whereas 57.8% has come from strategic programs. Open and strategic funding have both increased in similar magnitude and trajectories over this period and almost converge in the final year of the dataset.

### PI distribution

**Fig. 4:**
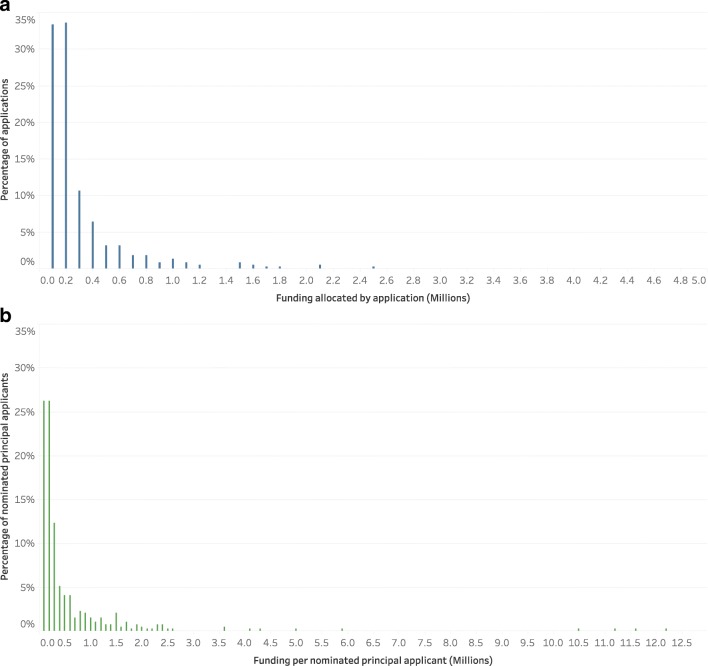
**a** Distribution of CIHR Funding: amount of funding received for global health research by funding application, 2000–2016. **b** Distribution of CIHR funding: amount of funding received for global health research by principal investigator, 2000–2016

A close analysis of operating grants reveals several important distribution patterns. The collective share of grants awarded to the 20 PIs who received the most global health research funding over the 15-year period totals $115 million—one third of all global health research funding (see Table 3). The most highly funded PI, Francis Plummer (University of Manitoba), received $12.1 million in global health research funding over this 15-year period, and the 20th most highly funded PI, Valéry Ridde (Université de Montréal), received $2.7 million. Figure 4a, b show the distribution of research funding by PI and by successful funding application. Of the 1584 funded applications, 60.0% were awarded less than $200,000 and 31.3% were awarded less than $100,000. Across 927 NPIs, 53.3% were awarded less than $200,000 and 30.0% were awarded less than $100,000.

**Table 3:** The 20 principal investigators receiving the most CIHR funding for global health research, 2000–2016 (adjusted for inflation to 2015 dollars)

	Name	Institution	Institute research area	Total
1	Francis A Plummer	University of Manitoba	Infection and immunity	$12,129,036
2	Geoffrey T Fong	University of Waterloo	Population and public health	$11,509,640
3	Kevin C Kain	University of Toronto	Infection and immunity	$11,145,183
4	Marc Ouellette	Université Laval	Infection and immunity	$10,479,514
5	André Lamy	McMaster University	Circulatory and respiratory health	$7,112,285
6	Silvia M Vidal	McGill University	Genetics	$5,837,121
7	Pierre Fournier	Hôpital Sainte-Justine (Montréal)	Human development, child, and youth health	$5,676,593
8	Richard Menzies	McGill University Health Centre	Population and public health	$5,164,180
9	Keith R Fowke	University of Manitoba	Infection and immunity	$4,902,522
10	Edward J Mills	University of Ottawa	Infection and immunity	$4,215,812
11	Peter S Tugwell	University of Ottawa	Population and public health	$4,192,593
12	Jerry M Spiegel	University of British Columbia	Population and public health	$4,032,251
13	Ronald Labonté	University of Ottawa	Population and public health	$4,019,371
14	Annalee Yassi	University of British Columbia	Population and public health	$3,975,475
15	Stephen Moses	University of Manitoba	Population and public health	$3,726,169
16	Daniel Gaudet	Université de Montréal	Population and public health	$3,534,627
17	Michel Alary	Université Laval	Infection and immunity	$3,515,023
18	Michel G Bergeron	Université Laval	Infection and immunity	$3,400,055
19	Banthia Nemkumar	University of British Columbia	Health services and policy research	$3,355,681
20	Valéry Ridde	Université de Montréal	Population and public health	$2,689,547

### Sex

Males predominate among the 20 most heavily funded global health PIs. Only three of the 20 PIs are female and rank 6th, 14th, and 19th, respectively. Of the 927 NPIs who received global health funding, 53.0% were male and 45.9% were female. Among the 1584 funded applications, 57.1% of the NPIs were male and 41.5% were female. Male PIs received approximately two thirds of both the open competitions (67.3%) and the priority competitions (61.0%), whereas female PIs received two thirds (66.3%) of global health training and career support awards. Female researchers have received more grants for global health research on gender and health (76.5%), Indigenous Peoples’ health (75.0%), and health services and policy research (58.2%), while male researchers have received more grants related to circulatory and respiratory health (87.5%), neurosciences, mental health and addiction (87.2%), musculoskeletal health and arthritis (81.8%), genetics (76.5%), infection and immunity (68.6%), and aging (65.0%).

### Infectious diseases

**Fig. 5:**
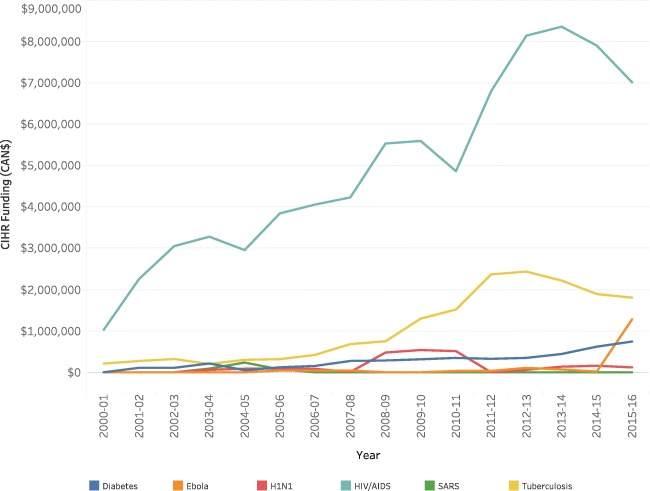
CIHR funding for global health research by infectious disease, 2000–2016

We analyzed trends in funding patterns for HIV/AIDS and tuberculosis over time, and following major infectious disease outbreaks of SARS, H1N1 influenza, and Ebola between 2000 and 2016 (Fig. 5). HIV/AIDS research funding far surpassed SARS, H1N1 influenza, Ebola, and tuberculosis. There was a small increase in SARS funding between FY2003/2004 and FY2005/2006 which then flat-lined thereafter. H1N1 influenza funding rose sharply in the three years following the virus’s 2009 global outbreak. Funding for HIV/AIDS and tuberculosis research both increased sharply after 2008. Global diabetes research was analyzed and included in the figure to serve as a baseline for comparison, as we did not expect diabetes funding to have changed in response to an external threat but only to have slowly increased over the period—which is indeed the case.

## Discussion

### Principal findings

After dramatic increases throughout the early 2000s, CIHR’s global health research funding plateaued in the early 2010s along with the agency’s overall budget. Canada’s position as an important middle power means that it will always face research funding constraints compared with larger jurisdictions such as Europe, the UK, and the US. As a middle power, one potentially effective strategy to maximize impact considering these constraints would be to prioritize funding based on the country’s existing strengths in global health research and the unique contributions it can make to existing national or global priorities based on comparative advantages (Cartier et al. 2018).

The marked increase in global health research funding from CIHR signals growing interest and strength among Canadian researchers in this area. CIHR funding over the course of the past 15 years has been concentrated in the Institute research areas of infection and immunity and population and public health. Nearly half of CIHR’s global health research funding during this period focused on health equity. Health equity was also one of the fastest growing focus areas, suggesting this might be an existing strength and comparative advantage upon which Canada can further build specialized leadership. Data on distribution of funds among applications and PIs paints a mixed picture of the degree to which funding is concentrated among top researchers or driven by many smaller applications. While the distribution of research funding shows 917 smaller awards (60.0%) less than C$200,000 each, 20 researchers (2.2%) account for one third of CIHR’s global health research funding.

The segmented breakdown of research funding across the different categories reveals changes over time. The share of biomedical funding has dipped in both absolute and relative terms in recent years, although it still represents more than one third of total funding overall. Conversely, the share of funding for population health research has increased steadily and eclipsed biomedical funding by increasingly greater margins starting in FY2012/2013. By FY2015/2016, annual population health research funding was nearly double that of biomedical research funding. The rapid increase of population health research over the last decade coincides with increased recognition for the broader social determinants of health in global health.

It is perhaps unsurprising that infection and immunity and population and public health are currently the top Institute research areas supported by CIHR’s global health research funding, as global health has historically focused on infectious diseases and because CIHR’s Institute of Population and Public Health (IPPH) has nurtured global health as a priority since its first strategic plan. IPPH is also CIHR’s largest financier of strategic priority-driven global health research initiatives and has overseen large collaborative investments, including the GACD and IMCHA programs with IDRC (Health equity matters 2015). While most aspects of health research are relevant to global health, some lend more easily to an explicit focus on global health than others. For example, the scope of infection and immunity and population and public health are often transnational, equity-focused, and targeted to reach populations facing conditions of marginalization both in LMICs and across multiple international settings. CIHR-funded research for Indigenous Peoples’ health has mostly been domestic in focus, with the relevant Institute’s mandate being to “improve and promote the health of First Nations, Inuit and Métis peoples in Canada”, whereas IPPH and CIHR’s Institute of Infection and Immunity have defined mandates that are global in scope (see Table 1).

While CIHR’s open investigator-driven competitions account for approximately 70% of annual expenditures, only 42.2% of global health research funding comes from this envelope. This discrepancy suggests the field depends upon continued funding from strategic priority-driven competitions and needs to be supported towards benefiting more greatly from CIHR’s open investigator-driven competitions. This discrepancy also highlights the need for peer-review committees to be appropriately structured, staffed, and supportive of global health research (Hoffman et al. 2018a; Behdinan et al. 2018; Hoffman et al. 2018b), and for harmful myths such as “CIHR doesn’t fund global health research” to be fully debunked in peer-reviewer training or by committee chairs during face-to-face meetings (Hoffman 2010).

However, global health research funding levels from the two types of competitions—open and strategic—almost achieved parity in FY2015/2016. This seems to be driven by steady increases in global health research funding from open investigator-driven competitions when preceded by investment in strategic priority-driven competitions, which suggests the level of CIHR’s strategic investments in global health research may determine the size of the field. Framed another way, Canada’s collective contributions to global health research may depend in large part on continued CIHR strategic investments that are ideally long-term and stable in nature.

The fact that 20 PIs collectively received one third of CIHR’s global health research funding may initially suggest that funds are too concentrated among a small group of individuals. However, a clear majority of funds are disbursed through smaller grants, as 53.3% of PIs hold grants less than $200,000, and two thirds of those PIs hold grants less than $100,000. Most highly funded researchers received both larger and smaller grants. This concentration of funds may alternatively reflect a deliberate strategy to prioritize excellence in research as opposed to a wider distribution of funds across more PIs. That said, CIHR funded 927 unique PIs from 1584 grants, which means almost two thirds of global health research grants have been awarded to PIs who only hold one global health research grant. A network analysis would reveal the extent to which CIHR’s top-funded PIs collaborate with other established researchers or with earlier career researchers—thereby further distributing access to global health research funds across the country.

### Strengths and weaknesses

There are four main strengths of this study. First, the analysis is based on a novel administrative dataset from CIHR. It is the first known analysis of the global health research portion of CIHR’s research investments. This analysis provided both a precise estimate of CIHR’s annual funding for global health research and a fine-grained segmented analysis of the shifts within funding categories over 15 fiscal years. Second, the data were independently validated twice: first by staff at CIHR’s IPPH and International Relations Team and second by our research team to minimize false positives. Third, steps were taken to increase the comparability of grants between years by adjusting for inflation and standardizing grant values to 2015 Canadian dollars. Fourth, this analysis is particularly informative for CIHR’s global health research strategy—which is especially important as CIHR is Canada’s largest health research funder broadly and for Canada-based global health researchers specifically. Our analysis of the CIHR dataset thus serves as a starting point for further investigation and provides a necessary benchmark against which future analyses can be compared.

There are three main limitations of this study. First, there is no standardized keyword classification scheme at CIHR. A search yielded more than 4700 unique keywords from the global health research dataset alone. This made further analysis on topics that would require information from the keyword metadata extremely labour-intensive due to risks of double-counting. For example, when analyzing the infectious disease research funding data, HIV/AIDS research was found to be coded under multiple variations: “HIV”, “AIDS”, “human immunodeficiency virus”, including their French-language equivalents of “VIH”, “SIDA”, and “virus de l'immunodéficience humaine”. Grants could also support projects involving more than one infectious disease; accordingly, double-counting could not be avoided (whereas all other analyses in this study were conducted using mutually exclusive categories to avoid any double-counting). The infectious diseases analysis therefore should be viewed as a preliminary analysis of trends rather than accurate magnitude. Future analyses would benefit from a standardized keyword classification scheme currently being considered by CIHR for its database. Second, the CIHR grants dataset had some missing information in two categories—institute research area and research theme—as they were both self-declared by grant applicants with options to be omitted. Data quality would be improved for these categories if CIHR required responses to them. Third, there was no feasible way to check CIHR’s funding database for false negatives, meaning there could theoretically be additional global health research grants within CIHR’s funding portfolio if CIHR’s IPPH or Strategic Partnerships and International Relations Team accidentally made mistakes when classifying grants as global health research.

### Future research directions

Future research would benefit from enhanced metadata collection by CIHR, a standardized keyword classification scheme, and more granular administrative data on investigators’ collaborations, career stage, age, gender, ethnicity, language, training, degrees, disciplines, and research specialization. It would also be helpful for CIHR to include new data categories for each grant, including the four foci of global health research. Ideally, CIHR could include more granular classifications within each focus area so that funded studies could be further analyzed, such as according to whether they aim to identify problems, test solutions, and/or develop methods.

Future research would also benefit from similar analyses of data from other major research funders in Canada, such as GCC and IDRC, as well as international funders such as the Bill & Melinda Gates Foundation and the Wellcome Trust to provide a fuller picture of the world’s global health research funding landscape. A data-driven analysis of global health research funding across all funders in Canada and globally has the potential to guide evidence-informed policymaking in this area that better aligns disparate funding activities towards unified strategies for global health research (Fafard and Hoffman 2018; Hoffman et al. 2009).

## Electronic supplementary material

ESM 1 (XLSX 1.45 mb)

ESM 2 (DOCX 17 kb)

ESM 3 (DOCX 22 kb)
