# Supplementary material for: Systematic analysis of global health research funding in Canada, 2000–2016
Source: Can J Public Health. 2019 Nov 6;111(1):80–95. doi: 10.17269/s41997-019-00247-8 (PMC7046862; doi:10.17269/s41997-019-00247-8)
Supplement: Supplementary file 2 — (DOCX 17 kb) [file 41997_2019_247_MOESM2_ESM.docx]

**Appendix 2. List of keywords used to retrieve CIHR’s global health research grants, 2015/16**

| #AFGHANI#;#AFGHAN#;#AFGHANS#;#AFGHANE#;#AFGHANES#;#AFRICA#;#AFRIQUE#;#AID#;#AMAZON#;#AMAZONIE#;#ARGENTINA#;#ARGENTINE#;#ASIA#;#ASIE#;#BANGKOK#;#BANGLADESH#;#BELARUS#;#B…LARUS#;#BELIZE#;#BENIN#;#B…NIN#;#BHUTAN#;#BHOUTAN#;#BOLIVIA#;#BOLIVIE#;#BOLIVIAN#;#BOLIVIEN#;#BOLIVIENS#;#BOLIVIENNE#;#BOLIVIENNES#;#BOTSWANA#;#BRAZIL#;#BR…SIL#;#BURKINA FASO#;#BURMESE-THAI#;#BIRMANO-THAœLANDAIS#;#BIRMANO-THAœLANDAISE#;#BIRMANO-THAœLANDAISES#;#BURUNDI#;#CAMBODIA#;#CAMBODGE#;#CAMEROON#;#CAMEROUN#;#CHILE#;#CHILI#;#CHINA#;#CHINE#;#CHINESE#;#CHINOIS#;#CHINOISE#;#CHINOISES#;#COLLABORATION INTERNATIONALE#;#COLLABORATIVE INTERNATIONAL#;#COLLABORATIF INTERNATIONAL#;#COLOMBIA#;#COLOMBIE#;#COMORES#;#CONGO#;#COSTA RICA#;#C‘TE D'IVOIRE#;#CUBA#;#CUBAN#;#CUBAIN#;#CUBAINS#;#CUBAINE#;#CUBAINES#;#D…VELOPPEMENT INTERNATIONAL#;#DOMINIQUE#;#EAST-INDIAN#;#INDIEN#;#INDIENS#;#INDIENNE#;#INDIENNES#;#ECUADOR#;#…QUATEUR#;#EGYPT#;#…GYPTE#;#EL SALVADOR#;#ESSENTIAL MEDICINE#;#M…DICAMENT ESSENTIEL#;#ETHIOPIA#;#…THIOPIE#;#ETHIOPIAN#;#…THIOPIEN#;#…THIOPIENS#;#…THIOPIENNE#;#…THIOPIENNES#;#FAIBLE REVENU#;#FILIPINO#;#PHILIPPIN#;#PHILIPPINS#;#PHILIPPINE#;#PHILIPPINES#;#GAMBIA#;#GAMBIE#;#GEORGIA#;#G…ORGIE#;#GHANA#;#GLOBAL ACCESS#;#ACC»S GLOBAL#;#GLOBAL COLLABORATIONS#;#COLLABORATIONS MONDIALES#;#GLOBAL HEALTH#;#SANT… MONDIALE#;#GLOBAL IMPACT#;#IMPACT MONDIAL#;#GLOBAL MEDICINE#;#M…DECINE MONDIALE#;#MARCH… MONDIAL DES M…DICAMENTS#;#GLOBAL PARTNERSHIP#;#PARTENARIAT MONDIAL#;#GLOBAL POLICIES#;#POLITIQUES MONDIALES#;#GLOBAL POLICY#;#POLITIQUE MONDIALE#;#GLOBAL POVERTY#;#PAUVRET… DANS LE MONDE#;#GLOBAL SOUTH#;#H…MISPH»RE SUD#;#PAYS DU SUD#;#GLOBAL TOBACCO#;#TABAGISME DANS LE MONDE#;#…PID…MIE MONDIALE DE TABAGISME#;#LUTTE MONDIALE AU TABAGISME#;#GLOBALIS#;#GLOBALIZ#;#GUATEMALA#;#GUAT…MALA#;#GUIN…E#;#GUYANA#;#HAITI#;#HAœTI#;#HONDURAS#;#INDIA#;#INDE#;#INDONESIA#;#INDON…SIE#;#INDONESIAN#;#INDON…SIEN#;#INDON…SIENS#;#INDON…SIENNE#;#INDON…SIENNES#;#INTERNATIONAL COLLABORATION#;#COLLABORATION INTERNATIONALE#;#INTERNATIONAL COLLABORATIVE#;#INTERNATIONALE COLLABORATIVE#;#COLLABORATIVE INTERNATIONALE#;#INTERNATIONAL COLLABORATORS#;#COLLABORATEURS INTERNATIONAUX#;#COLLABORATRICES INTERNATIONALES#;#INTERNATIONAL DEVELOPMENT#;#D…VELOPPEMENT INTERNATIONAL#;#INTERNATIONAL HEALTH#;#SANT… INTERNATIONALE#;#INTERNATIONAL STUDIES#;#…TUDES INTERNATIONALES#;#INTERNATIONAL STUDY#;#…TUDE INTERNATIONALE#;#IRAN#;#IRAQ#;#JAMAICA#;#JAMAœQUE#;#JORDAN#;#JORDANIE#;#KENYA#;#KENYANS#;#K…NYAN#;#K…NYANS#;#K…NYANE#;#K…NYANES#;#KYRGYZSTAN#;#KIRGHIZISTAN#;#LAOS#;#LATIN AMERICA#;#AM…RIQUE LATINE#;#LESOTHO#;#LMIC#;#PRFI#;#LOW AND HIGH INCOME#;#REVENU FAIBLE ET …LEV…#;#LOW AND MIDDLE INCOME#;#REVENU FAIBLE ET INTERM…DIAIRE#;#LOW AND MIDDLE-INCOME#;#¿ REVENU FAIBLE ET INTERM…DIAIRE#;#LOW- AND MIDDLE-INCOME#;#¿ REVENU FAIBLE ET MOYEN#;#LOW DISPOSABLE INCOME#;#REVENU DISPONIBLE FAIBLE#;#LOW INCOME#;#REVENU FAIBLE#;#FAIBLE REVENU#;#LOW- INCOME#;#¿ REVENU FAIBLE#;#¿ FAIBLE REVENU#;#LOW INCOME COUNTRIES#;#PAYS ¿ REVENU FAIBLE#;#LOW INCOME COUNTRY#;#PAYS ¿ FAIBLE REVENU#;#LOW LEVELS OF INCOME#;#REVENU FAIBLE#;#LOW LITERACY AND INCOME#;#FAIBLE TAUX DíALPHAB…TISATION ET DE REVENU#;#LOW OR LOW-MIDDLE INCOME#;#REVENU FAIBLE OU REVENU FAIBLE ¿ INTERM…DIAIRE#;#LOW OR MIDDLE INCOME#;#REVENU FAIBLE OU INTERM…DIAIRE#;#LOW- OR MIDDLE-INCOME#;#¿ REVENU FAIBLE OU INTERM…DIAIRE#;#LOW-AND MIDDLE-INCOME#;#¿ REVENU FAIBLE ET INTERM…DIAIRE#;#LOW-AND-MIDDLE INCOME COUNTRIES#;#PAYS ¿ REVENU FAIBLE ET INTERM…DIAIRE#;#LOW-INCOME#;#FAIBLE REVENU#;#LOW-MIDDLE INCOME COUNTRY#;#PAYS ¿ REVENU FAIBLE ¿ INTERM…DIAIRE#;#LOW-TO-MIDDLE INCOME COUNTRIES#;#PAYS ¿ REVENU FAIBLE ¿ MOYEN#;#MADAGASCAR#;#MALAWI#;#MALAYSIA#;#MALAISIE#;#MALI#;#MAURITANIE#;#MAURITIUS#;#MAURICE#;#MAYAN#;#MAYA#;#MAYAS#;#MAYOTTE#;#M…DECINE TROPICALE#;#MEXICAN#;#MEXICAIN#;#MEXICAINS#;#MEXICAINE#;#MEXICAINES#;#MEXICO#;#MEXIQUE#;#MIDDLE INCOME COUNTRIES#;#PAYS ¿ REVENU INTERM…DIAIRE#;#MIDDLE-INCOME COUNTRIES#;#PAYS ¿ REVENU MOYEN#;#MONDIALISATION#;#MONGOLIA#;#MONGOLIE#;#MOZAMBICAN#;#MOZAMBICAIN#;#MOZAMBICAINS#;#MOZAMBICAINE#;#MOZAMBICAINES#;#MOZAMBIQUE#;#NEPAL#;#N…PAL#;#NIGER#;#PAKISTAN#;#PAN-AFRICAN#;#PANAFRICAIN#;#PANAFRICAINS#;#PANAFRICAINE#;#PANAFRICAINES#;#PARAGUAY#;#PARTENARIAT NORD-SUD#;#PAYS ¿ FAIBLES RESSOURCES#;#PAYS EN D…VELOPPEMENT#;#PAYS EN VOIE DE D…VELOPPEMENT#;#PERU#;#P…ROU#;#PHILIPPINES#;#PRFI#;#PRFM#;#PUNJABI#;#RESEARCH FOR DEVELOPMENT#;#RECHERCHE POUR LE D…VELOPPEMENT#;#RWANDA#;#SAHARA#;#SAINT VINCENT#;#SAINT-VINCENT#;#SANT… INTERNATIONALE#;#SANT… MONDIALE#;#SENEGAL#;#S…N…GAL#;#SERBIA#;#SERBIE#;#SEYCHELLES#;#SOMALI#;#SOMALIEN#;#SOMALIENS#;#SOMALIENNE#;#SOMALIENNES#;#SOUTH AFRICA#;#AFRIQUE DU SUD#;#SRI LANKA#;#SRILANKA#;#SUB-SAHARAN#;#SUBSAHARIEN#;#SUBSAHARIENS#;#SUBSAHARIENNE#;#SUBSAHARIENNES#;#SUDAN#;#SOUDAN#;#SYRIAN#;#SYRIEN#;#SYRIENS#;#SYRIENNE#;#SYRIENNES#;#TAMIL#;#TAMOUL#;#TANZANIA#;#TANZANIE#;#THAILAND#;#THAœLANDE#;#THIRD WORLD#;#TIERS MONDE#;#TRINIDAD#;#TRINIT…#;#TROPICAL DISEASE#;#MALADIE TROPICALE#;#TROPICAL MEDICINE#;#M…DECINE TROPICALE#;#TUNISIE#;#TURK#;#TURC#;#TURCS#;#TURQUE#;#TURQUES#;#UGANDA#;#OUGANDA#;#UKRAINE#;#URUGUAY#;#VENEZUELA#;#V…N…ZUELA#;#VIETNAM#;#VIETNAMESE#;#VIETNAMIEN#;#VIETNAMIENS#;#VIETNAMIENNE#;#VIETNAMIENNES#;#WITHOUT BORDERS#;#SANS FRONTI»RES#;#YEMEN#;#Y…MEN#;#ZAMBIA#;#ZAMBIE#;#ZIMBABWE#;#DEVELOPING COUNTRY#;#PAYS EN D…VELOPPEMENT#;#DEVELOPING COUNTRIES#;#PAYS EN VOIE DE D…VELOPPEMENT#;#SUSTAINABLE DEVELOPMENT#;#D…VELOPPEMENT DURABLE#;#LOW_INCOME#;#FAIBLE_REVENU#;#MIDDLE_INCOME#;#REVENU_INTERM…DIAIRE#;#REVENU_MOYEN# |
| --- |
