# Supplementary material for: Systematic analysis of global health research funding in Canada, 2000–2016
Source: Can J Public Health. 2019 Nov 6;111(1):80–95. doi: 10.17269/s41997-019-00247-8 (PMC7046862; doi:10.17269/s41997-019-00247-8)
Supplement: Supplementary file 3 — (DOCX 22 kb) [file 41997_2019_247_MOESM3_ESM.docx]

Appendix 3. Highest CIHR funded research projects by focus area, 2000-2016.

| **Focus area** | **Current NPI** | **Institution** | **Grant program** | **Project title** | **Abstract** | **Effective date** | **Total disbursed^[[1]](#footnote-1)^** |
| --- | --- | --- | --- | --- | --- | --- | --- |
| Globalization | Lamy, André | McMaster University | Randomized Controlled Trials | CABG off or on pump revascularization study (coronary) | Coronary artery disease remains a major cause of death in Canada and consumes a significant portion of our health care budget. Coronary artery bypass graft surgery (CABG) is a well-recognized treatment of advanced coronary artery disease. Conventional CABG is performed with the help of a cardiopulmonary bypass circuit (CPB), commonly called the pump. While cardiopulmonary bypass allows the surgeon to perform the delicate surgery it has been associated with significant complications such as cardiac ischemia, stroke, neuro-cognitive dysfunction and renal dysfunction. In order to avoid these deleterious consequences, cardiac surgeons have designed new heart stabilizers to allow them to perform CABG on a beating heart without using cardiopulmonary bypass (off-pump CABG) and reduce costs of CABG surgery. The CORONARY trial is a prospective multinational randomized trial comparing the new off-pump CABG technique (beating heart surgery) vs. the conventional on-pump CABG surgery. This trial will recruit 4700 patients from 100 cardiac surgery centers in North America, South America, Western Europe Eastern Europe Middle East and Asia. The co-primary outcomes are a composite of total mortality, MI, stroke, renal failure and repeat revascularization at 30 days and 5 years. The secondary outcome is a cost-effectiveness analysis. We are confident to demonstrate an improvement in the safety of CABG surgery and reduce costs in the delivery of cardiac services. | 01/10/2007 | $7,112,284.54 |
| Health Equity | Fong, Geoffrey T | University of Waterloo (Ontario) | Operating Grant | The International Tobacco Control Policy Evaluation Project: Evaluating the impact of the WHO Framework Convention on Tobacco Control with an Emphasis on Low and Middle Income Countries | Tobacco use has been identified by the World Health Organization (WHO) as the world's number one preventable cause of death. In the 21st Century, it is projected that one billion people may die of tobacco use, and the toll will be greatest in low- and middle-income countries (LMICs). In 2003, the 192 WHO countries adopted the Framework Convention on Tobacco Control (FCTC), the first-ever health treaty, which specifies a broad set of tobacco control policies that the FCTC Parties (now numbering 172 countries) must implement, e.g., enhanced warning labels, bans/restrictions on advertising/promotion, increased taxation, and smokefree laws. The International Tobacco Control Policy Evaluation Project (ITC Project) is a Canada-led collaboration of leading tobacco control researchers across 22 countries (over 70% of the world's tobacco users) whose mission is to conduct rigorous evaluation of the impact of FCTC policies; it is the only international effort designed to evaluate the FCTC, that is, the only research program that focuses on whether the treaty is having its intended effects. The ITC Project uses state-of-the-art survey methods to answer key questions: Do graphic warnings lead to increased motivation to quit? Are smoke-free laws effective in reducing smoking in public places? Do higher taxes on cigarettes lead to shifts to other tobacco forms (e.g. bidis in Bangladesh) rather than to quitting? We will also examine how policy effects may vary across the 22 countries. The ITC Project has built an extensive research-to-action network in which we disseminate/transfer our findings rapidly in each country to key contacts in government, the research community, and Civil Society, which has led to significant policy changes in several countries, including Canada. The ITC Project has become an effective global system for generating, disseminating, and promoting the utilization of knowledge in the fight against the growing global tobacco epidemic, particularly in LMICs. | 01/10/2011 | $6,899,388.71 |
| Transnational Risks | Plummer, Francis A | University of Manitoba | Training Grant - LOI | Using mathematical modeling and health economics to evaluate and optimize infectious disease prevention strategies | N/A | 01/03/2002 | $2,201,029.38 |
| Tropical Diseases | Vidal, Silvia M | McGill University | CIHR Team Grant Program | CIHR Team in Mutagenesis and Infectious Diseases | Although large strides have recently been taken in the characterization of innate response to microbes, deficit in host resistance to infection remains a primordial problem. Host genetic factors play an important role in the onset, progression, and ultimate outcome of infection with pathogens. To effectively address the challenge of emerging and established infectious diseases in human health, we propose the creation of a large multidisciplinary team of Canadian scientists and international collaborators to systematically identify and characterize mouse genes conferring host resistance against infectious pathogens of global relevance: malaria, influenza and enterobacteria. Ultimately, the mutated genes identified in mice will be tested as candidates for infectious disease susceptibility among well-defined affected human populations and will serve as a substrate for biochemical and medical chemistry studies. | 01/10/2008 | $4,049,164.72 |

1. In 2015 Canadian Dollars [↑](#footnote-ref-1)
